# Early smallpox vaccine manufacturing in the United States: Introduction of the “animal vaccine” in 1870, establishment of “vaccine farms”, and the beginnings of the vaccine industry

**DOI:** 10.1016/j.vaccine.2020.05.037

**Published:** 2020-06-19

**Authors:** José Esparza, Seth Lederman, Andreas Nitsche, Clarissa R. Damaso

**Affiliations:** a Institute of Human Virology, University of Maryland School of Medicine, Baltimore, MD, USA; b Tonix Pharmaceuticals, New York, NY, USA; c Centre for Biological Threats and Special Pathogens 1 – Highly Pathogenic Viruses & German Consultant Laboratory for Poxviruses & WHO Collaborating Centre for Emerging Infections and Biological Threats, Robert Koch Institute, Berlin, Germany; d Instituto de Biofísica Carlos Chagas Filho, Universidade Federal do Rio de Janeiro, Rio de Janeiro, Brazil

**Keywords:** Diphtheria antitoxin, Smallpox, Smallpox vaccine, Vaccine farm, Vaccine industry, Vaccinia

## Abstract

For the first 80–90 years after Jenner’s discovery of vaccination in 1796, the main strategy used to disseminate and maintain the smallpox vaccine was arm-to-arm vaccination, also known as Jennerian or humanized vaccination. A major advance occurred after 1860 with the development of what was known as “animal vaccine”, which referred to growing vaccine material from serial propagation in calves before use in humans. The use of “animal vaccine” had several advantages over arm-to-arm vaccination: it would not transmit syphilis or other human diseases, it ensured a supply of vaccine even in the absence of the spontaneous occurrence of cases of cowpox or horsepox, and it allowed the production of large amounts of vaccine. The “animal vaccine” concept was introduced in the United States in 1870 by Henry Austin Martin. Very rapidly a number of “vaccine farms” were established in the U.S. and produced large quantities of “animal vaccine”. These “vaccine farms” were mostly established by medical doctors who saw an opportunity to respond to an increasing demand of smallpox vaccine from individuals and from health authorities, and to make a profit. The “vaccine farms” evolved from producing only smallpox “animal vaccine” to manufacturing several other biologics, including diphtheria- and other antitoxins. Two major incidents of tetanus contamination happened in 1901, which led to the promulgation of the Biologics Control Act of 1902. The US Secretary of the Treasury issued licenses to produce and sell biologicals, mainly vaccines and antitoxins. Through several mergers and acquisitions, the initial biologics licensees eventually evolved into some of the current major American industrial vaccine companies. An important aspect that was never clarified was the source of the vaccine stocks used to manufacture the smallpox “animal vaccines”. Most likely, different smallpox vaccine stocks were repeatedly introduced from Europe, resulting in polyclonal vaccines that are now recognized as “variants” more appropriately than “strains”. Further, clonal analysis of modern “animal vaccines” indicate that they are probably derived from complex recombinational events between different strains of vaccinia and horsepox. Modern sequencing technologies are now been used by us to study old smallpox vaccine specimens in an effort to better understand the origin and evolution of the vaccines that were used to eradicate the smallpox.

## Highlights

• Manufacturing of the smallpox vaccine at the end of the 19th century was the beginning of the vaccine industry in the United States.
• There is not information regarding the stocks or seed viruses used to manufacture those early vaccines.
• Historical information, will allow a better understanding of the origin and evolution of the vaccine used to eradicate smallpox.

## 1 Introduction

In his famous “Inquiry” published in 1798, Edward Jenner described the principles of cowpox inoculation or vaccination against smallpox [1]. In the same small book he also reported that the vaccine could be serially transmitted from human to human, suggesting a practical system based on “arm-to-arm” vaccination [2]. That procedure, known as Jennerian or humanized vaccination, was practiced extensively during the following 80–90 years after the discovery of vaccination, for the maintenance, propagation and distribution of the smallpox vaccine. The vaccine could also be transported adsorbed to the points of small lancets (made of ivory, silver or gold), between small glass plates containing desiccated vaccine sealed with wax, in long threads with desiccated vaccine, or using dried crusts or scabs. Those procedures were cumbersome and unreliable. Very often the vaccine became inactivated on transit, or the vaccine stocks were lost because of interruptions in the arm-to-arm chains of transmission of the vaccine. Consequently, there was a frequent need for reintroducing vaccine material either from vaccinated children from other localities, or directly from newly discovered cases of naturally occurring cowpox or horsepox [2], [3]. An additional problem of the arm-to-arm passage of the vaccine was the unintentional transmission of human diseases, especially syphilis. The above difficulties were obviated with the introduction of the so-called “Animal Vaccine”.

## 2 Animal vaccine

**“**Animal vaccine“ referred to the vaccine that was obtained directly from a cowpox or horsepox lesion on cows and serially propagated in calves, without undergoing human passages before it was ultimately used to vaccinate humans [4], [5]. “Retrovaccination” was a different procedure by which the humanized vaccine was passed one or more times in calves with the belief that doing so enhanced its potency. The use of “animal vaccine” had several advantages over arm-to-arm vaccination: it would not transmit syphilis or other human diseases, it ensured a constant supply of vaccine even in the absence of occurrence spontaneous cases of cowpox or horsepox, and it allowed for the production of large amounts of vaccine.

Animal vaccination started in Naples, Italy, in 1840 when Giuseppe Negri succeeded in continuously carrying the vaccine in calves. The use of animal vaccination was confined almost exclusively to Italy until the method was extensively discussed in 1860 at a Medical Congress held at Lyon, France. Among the participants at the meeting was Ernest Chambon, still a medical student at that time, who convinced one of his friends, Gustave Lanoix, to try the procedure [6]. Lanoix visited Naples to learn the technique, returning back to Paris with an inoculated heifer. Chambon and Lanoix then created in Paris in 1864 a private Institute of Animal Vaccines [7]. The virus subsequently used at the Paris institute was replaced by the famous Beaugency lymph, which was a mixture of material obtained from spontaneous cases of cowpox discovered in 1866 at Beaugency in the Loire Valley and in the suburbs of Paris [5], [8].

Animal vaccination was rapidly adopted around the world, although arm-to-arm vaccination continued to be performed in parallel for some time, while the medical community debated the value of the “animal vaccine” in comparison with that of the humanized vaccine. Gradually “animal vaccine” was preferred to humanized vaccine, to the point where countries began to prohibit humanized vaccination. England was the last major country to prohibit the practice of humanized vaccination in 1898. The development of the “animal vaccine” technology, together with an ever increasing need to produce large number of vaccine doses demanded by the health authorities, led to the establishment of numerous “vaccination parks” (as they were usually called in Europe) or “vaccine farms” (as they were referred to in the United States) around the world, using in many cases pools of different vaccine stocks for the inoculation of calves. Animals were inoculated in multiple sites, from which material was collected when the lesions were judged to be “ripe” and the “pulp” was grounded in a mortar before suspended in diluent as the “vaccine lymph” [3]. The proliferation of vaccine parks with poor standards led a British contemporary author to complain that, “The country is flooded with cheap stuff made in Germany and elsewhere, of unknown nature or origin” [9].

## 3 Henry Austin Martin introduced the “animal vaccine” in the United States, which was followed by a proliferation of “vaccine farms”

Although smallpox vaccination was first introduced in the United States in 1800 [10], it was not until 1870 when “animal vaccine” was introduced by the Boston physician Henry Austin Martin [11]. Dr. Martin dispatched an agent to Paris to obtain the virus from Dr. Jean-Anne-Henri Depaul, Director of the Vaccination Services of the Paris Academy of Medicine. Depaul supplied him with vaccine from the 258th, 259th and 260th passage of his continuous series from the heifer of Beaugency [12], [13], [14]. The “animal vaccine” was received in New York in September 1870 and immediately used by Dr. Martin to inoculate a few heifers. Reportedly, the vaccine stock from Dr Martin was the one exclusively used in the Unites States for the following six years.

**Fig. 1:**
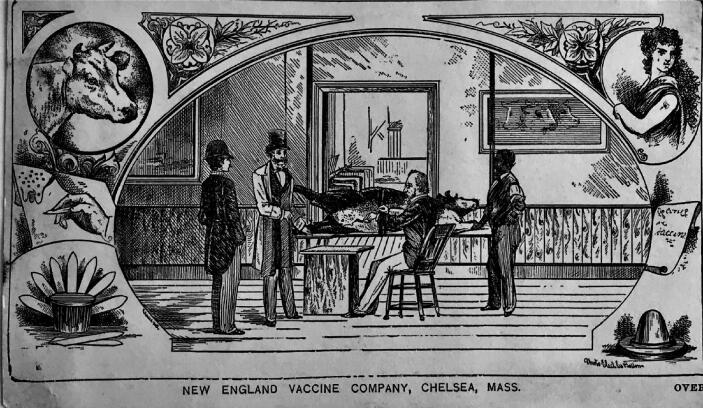
Advertisement card of a Vaccine Farm (New England Vaccine Company, Chelsea Station, Boston, Mass.; established in 1871). Card dated 1884. JE Collection.

As it was done in Europe, a large number of vaccine farms began to be established in the United States. Table 1 shows a list of the best known vaccine farms existing in the United States in 1897 [15]. However, many other vaccine farms, some of ephemeral existence, were established between the 1870 s and 1900. Most of the vaccine farms were initiated by medical doctors who saw an opportunity to respond to the ever increasing demand of smallpox vaccine from individuals and from health authorities and make a profit. The “vaccine farms” that were established in the United States at the end of the 19th century were in general small businesses, usually conformed by a small stable and an operating room used to harvest the vaccine from inoculated heifers. These “vaccine farms” were occasionally inspected by State health authorities that evaluated the general hygienic conditions of the stables and operating rooms, as well as the techniques used to inoculate the calves, to collect the vaccine material and to dispense it in capillaries or in any other system for vaccine delivery, such as ivory points. There were concerns about bacterial contamination of the vaccines, although most “vaccine farms” relied on “simple cleanliness of facilities and procedures”. Nevertheless, products from several “vaccine farms” were submitted to bacteriological analysis (basically, bacterial counts) and, if the results were good, they were used to advertise the quality of the vaccine. The presumed “quality” of the vaccine was mostly assessed by testimonials provided by users, based on the characteristics of the major cutaneous lesions induced after vaccination (the “take”) and, when possible, on empirical assessment of the apparent protective value of the vaccine against cases of smallpox in the community [16]. “Vaccines farms” widely advertised their products among the medical community and the general public (Fig. 1).

**Table 1:** Vaccine Farms in the United States (1897) [15]

Name	Location	Responsible(s)
The Franklin County Vaccine Farm	Franklin County, PA	John Seibert
The Jenner Vaccine Farm	Chambersburg, PA	L. F. Suesserott
The Pennsylvania Vaccine Company	Chambersburg, PA	M. M. McKnight & Co
The Lancaster County Vaccine Farms	Marietta, PA	H. M. Alexander
The National Vaccine Establishment	Chevy Chase, MD	Ralph Walsh, Frank Elgin
The Chicago Vaccine Stables	Harlem, Chicago, IL	E. A. Wood, K. Oakes
The Codman and Shurtleff Vaccine Farm	Stoughton, MA	Codman, Shurtleff
The Dr. F. C. Martin Vaccine Farm	Newton Centre, MA	Francis E. Martin
The New England Vaccine Company	Boston, MA	Cutler, Frisbie, Gains
The Missouri Vaccine Farm	St. Louis, MO	R. M. Higgins
The Columbia Vaccine	Columbia, MO	Woodson Moss, D. D. Moss
The Fond du Lac Vaccine Company	Fond du Lac, WI	E. B. Beeson
The Doctor Henry McNeel Company	Fond du Lac, WI	Henry McNeel
The Dr. H Welker Company	Milwaukee, WI	H. Welcker

Proprietors of some of the “vaccines farms” claimed to be using the Beaugency strain for the production of their “animal vaccine”. In November 1870, Dr Frank Pierce Foster of the New York Dispensary, commenced propagation of the virus obtained from Henry Austin Martin [17]. Dr Martin also provided the Beaugency stock to a “vaccine farm” established by Dr. E L Griffin of Fond du Lac, WI, although he accidentally lost the stock in 1880, obtaining a new stock from Brussels. Martin also provided the Beaugency virus to “vaccine farms” in New England. The Jenner Vaccine Farm at Chambersburg PA also claimed, as many others did, to be propagating the Beaugency strain directly imported from France. However, many other vaccine stocks were introduced from Europe during that time. The reality is that by the end of the 1890 s “vaccine farms” were not really concerned about the origin or pedigree of their vaccine stocks, which probably derived from multiple vaccines imported at different times from different European establishments.

At the time when the “vaccine farms” were initially established, the only existing vaccine was the one against smallpox, and during most of the 19th century the term “vaccine” was synonymous of “smallpox vaccine”. However, the decades of 1870 to 1900 saw a rapid development of the science of microbiology and the formulation of the germ theory of disease, especially by the work of Louis Pasteur, Robert Koch and their disciples [18]. With the development of the germ theory of disease a large number of specific microorganisms were identified as etiological agents of different human and animal diseases and in 1881 Louis Pasteur demonstrated the possibility of preventing anthrax with attenuated preparations of the *Bacillus anthracis*. To honor Jenner, in 1881 Louis Pasteur proposed at the 7th International Congress of Medicine held in London, to generalize the term “vaccination” to refer to all protective immunization procedures against any infectious diseases [19] and since then we talk about vaccines against different diseases. The second vaccine for human use was against rabies, developed by Pasteur in 1885.

Advances in microbiology were not restricted to the discovery of new pathogens or to the production of vaccines, but also to the development of passive protection by specific antibodies. In this regard, it is relevant to mention the case of diphtheria, which was a major cause of child mortality in the United States by the end of the 19th century. The etiological agent, *Corynebacterium diphtheria,* was isolated in 1883 by Friedrich Löffler, an associate of Robert Koch in Berlin. In 1890 Emil von Behring and Shibasaburo Kitasato, also working at the Robert Koch’s institute in Berlin, developed antitoxins against both tetanus and diphtheria, and in 1894 Emil Roux in Paris began the large scale production of diphtheria antitoxin in immunized horses. The diphtheria antitoxin provided an effective way to treat patients with diphtheria, and for that discovery von Behring received the inaugural Nobel Prize in Physiology or Medicine in 1901.

Those advances in microbiology, including the production of diphtheria antitoxin, were rapidly imported into the United States and incorporated in the portfolio of some of the most advanced “vaccine farms” in the country. By 1895 diphtheria antitoxin was being produced in horses, first by the New York City Board of Health and the Hygienic Laboratory of the Marine Hospital Service, and soon after by commercial organizations that also produced smallpox vaccines, such as Parke Davis and Co. and Mulford Laboratories [20].

Two unfortunate incidents happened in 1901 that changed the landscape of how relatively uncontrolled “vaccine farms” functioned in the United States. One was a cluster of 13 children who died of tetanus from a contaminated preparation of diphtheria antitoxin produced in a diseased horse by the St. Louis Board of Health. The second incident was the death of nine children in Camden, New Jersey, who developed tetanus after receiving contaminated smallpox vaccine [21], [22].

Those two events received much public attention, emphasizing the need to strengthen the regulatory oversight of the production of biological products. This led to the passage in 1902 of the Biologics Control Act that regulated “the sale of viruses, vaccines, serums, toxins, and analogous products”. The Act established a Board oversaw by the Secretary of the Treasury, with the authority to issue licenses to produce and sell biological products.

## 4 Major smallpox vaccine producers after the biologics Control Act of 1902

The purpose of the Biologics Act of 1902 was to control the purity and potency of biological products intended to cross state borders. Control was exerted by inspections, laboratory controls, and licenses. The licenses were issued by the Secretary of the Treasury for specific products, and could also be given to foreign manufacturers [23]. The manufacturing of biologics (particularly smallpox vaccine and diphtheria antitoxin) was an established business model, not only because of the existing private demand, but more importantly because of lucrative government contracts to supply vaccines to the public sector.

By 1903 13 licenses had been awarded, including to two foreign organizations, in France and Germany. Licenses numbers 1, 2, and 3, respectively, were given to three distinguished American companies: Parke Davis & Co. from Detroit, MI, H.K. Mulford from Philadelphia, PA, and Dr. H. M. Alexander & Co. from Marietta, PA. Over time, some of the licensed establishments changed names while others simply dropped off from the list when they failed to meet the necessary requirements. Table 2 shows some of the establishment licensed by 1912, focusing on those producing smallpox vaccine [24].

**Table 2:** Establishments licensed to manufacture smallpox vaccine in the United States (1912) [24]

License #	Name	Location
1	Parke, Davis & Co.	Detroit, MI
2	H. K. Mulford	Philadelphia, PA
3	Dr. H. M. Alexander & Co.	Marietta, PA
4	Fluid Vaccine Co.	Milwaukee, WI
5	The Slee Laboratories	Swiftwater, PA
8	The Cutter Laboratory	Berkeley, CA
14	Health Department of the City of New York	New York, NY
16	National Vaccine and Antitoxin Institute	Washington, DC

Two establishments were authorized to produce only smallpox vaccine (Fluid Vaccine Co. and the Slee Laboratories), whereas most were also authorized to produced diphtheria antitoxin (Parke Davis & Co, H. K. Mulford Co, Dr. H. M. Alexander, The Cutter Laboratories, and the Health Department of the City of New York). A few laboratories, especially Parke, Davis & Co. and H. K. Mulford, were authorized to produce many other biologicals, including antitetanic serum, antitubercle serum, antigonococcic sera, antityphoid sera, bacterial vaccines, tuberculins, antirabic serum and smallpox vaccine.

A short description of the licensed establishments would serve to illustrate the dynamics of the vaccine and antitoxin industry at the beginning of the 20th century in the United States, showing how the almost amateurish vaccine farms from the end of the 19th century began to evolve into the modern American vaccine industry. It is a complicated story of mergers and acquisitions and we will only focus on issues relevant to smallpox vaccines.

***Parke, Davis & Co.*** Its origin can be traced to a small drug manufacturing business established in Detroit in 1866. By 1874 their catalog listed a large number of products, many of them derived from ethnobotanical medicine. In the early 1890 s the company started to explore animal products as medicines, such as desiccated thyroid glands. When the development of diphtheria antitoxin was announced in Europe, the company immediately started its manufacture, with a product in clinical use in 1895. Two years later antistreptococcic and antitetanic serums were also in the market [25], [26]. Although not much publicity was given to the production of smallpox vaccine, it started in 1898. Warner-Lambert acquired Parke-Davis in 1970, which in turn was acquired by Pfizer in 2000.

***H. K. Mulford Co.*** Henry K. Mulford first established the company in the late 1880 s when he purchased a drugstore in Philadelphia, starting to produce a line of pharmaceutical preparations. In 1894 the company hired Joseph McFarland, a bacteriologist from the University of Pennsylvania, and by 1895H. K. Mulford Co. was commercially producing diphtheria antitoxin, reportedly the first private company to produce it [27], [28]. In 1896 the company moved to Glenolden, PA, and in 1898 entered the business of smallpox vaccine production. For that purpose it hired Dr. William Franklin Elgin, an experienced bacteriologist working at that time at a vaccine farm known as The National Vaccine Establishment in Washington, DC, which was managed by Dr. Ralph Walsh (see Table 1). [29], [30]. Soon H. K. Mulford Co. became one of the major smallpox vaccine producer in the United States, and also manufacturing a large number of vaccines and other biologics. In 1929 Mulford was acquired by the Baltimore-based Sharpe & Dohme, whose business was focused on prescription drugs and in 1953 Sharpe & Dohme merged with Merck.

***Dr H. M. Alexander& Co.*** Among the several vaccine farms operating in Pennsylvania at the end of the 19th century one of the major ones was founded in Marietta in 1882 by Dr. H. M. Alexander, with the original name of The Lancaster County Vaccine Farm (see Table 1) [31], [32], [33]. A veterinarian, Dr. Samuel H. Gilliland was hired to run the business. He married one of the Alexander daughters and in 1917 purchased the business, changing the name to The Gilliland Laboratories, which continued producing smallpox vaccines. In 1943 The Gilliland Laboratories was acquired by American Home Products. American Home Products itself had origins as a “vaccine farm”. In 1860 John Wyeth and Brother (Frank) established a drugstore in Philadelphia, and by 1886 they were operating a “model vaccine farm” in Chester County, PA [34], [35]. By the 1960 s Wyeth was the only manufacturer of smallpox vaccine in the United States. Their Dryvax was a freeze dried calf lymph smallpox vaccine supposedly derived from the New York City Board of Health (NYCBH) vaccine strain [36], licensed in 1944 and produced in the Marietta vaccine plant. In 2002, American Home Products was renamed Wyeth in recognition of its roots. Ultimately, in 2009 Wyeth was acquired by Pfizer.

***Fluid Vaccine Co.*** It was basically a one man operation derived from the Dr. H Welcker Company (vaccine farm) originally located in Milwaukee, WI, owned and operated by Dr. H Welcker (or Welker) (see Table 1). Reportedly they produced the first glycerinated liquid smallpox vaccine used in the United States [37]. Glycerol was introduced by Giuseppe Negri in Italy the 1840 s as vaccine diluent and the use of glycerol was popularized after 1892 when Monkton Copeman reported its use not only as a convenient liquid for suspension of the vaccine in terms of its clarity and viscosity, but more importantly because its bactericidal effect [38].

***The Slee Laboratories.*** Founded in 1911, this laboratory was the successor of the Pocono Biological Laboratories established in 1897 in Swiftwater, PA, by Dr. Richard M Slee, which in 1903 received the Biologics license number 4. Dr Slee had visited France to learn the techniques to produce glycerinated smallpox vaccines, which he started to produce in 1898 [39]. In 1907 Dr. Slee temporarily closed the Pocono Laboratories and moved to New York to direct the production of biological products at the Lederle Antitoxin Laboratory in Pearl River, NY. Lederle was acquired by American Home Products and the Pearl River facility even today manufactures the Pneumovax pneumococcal vaccine. He then returned to Swiftwater in 1911 to reopen the facilities with the new name of the Slee Laboratories, continuing the production of the smallpox vaccine as well as of a variety of biologicals for human and veterinary medicine, some of them produced for The Abbot Laboratories of Chicago [40]. In 1927 the Swiftwater facilities were leased to the Philadelphia based National Drug Company which the year before had hired Dr. H.K. Mulford as its Director of Research and Biological. The National Drug Company expanded their vaccine portfolio over the years, with new constructions at the Swiftwater site. From 1978 to 1989, the Swiftwater facility was operated by Connaught, the major vaccine manufacturer in Canada. In 1989 the Mérieux Institute acquired the Connaught Laboratories and eventually the Swiftwater facilities became part of Sanofi Pasteur [41].

***The Cutter Laboratory.*** Founded in 1897 by Edward Ahern Cutter in Fresno, California, as a bacteriological laboratory to conduct analysis of clinical samples. Very soon it started producing vaccines against anthrax, although their most important product was a vaccine against blackleg, a debilitating cattle disease caused by *Clostridium chauvei*. In 1903 their business moved to Berkeley, California, adopting the name of The Cutter Laboratory and began to produce smallpox vaccines and diphtheria antitoxin in addition to several veterinary products. Their first smallpox vaccine was sold in 1904 [42]. The Cutter Laboratories became one the first major pharmaceutical companies in the United States, producing a variety of vaccines and antitoxins, also having the merit of being the first pharmaceutical in the United States to use alum as a vaccine adjuvant. However, The Cutter Laboratories is mostly remembered today by the so-called “Cutter Incident”, when a poorly inactivated polio vaccine produced by the company in 1955 was implicated in causing at least 56 cases of paralytic polio and 5 deaths [43]. In 1974 The Cutter Laboratories were bought by Bayer Pharmaceuticals.

***Health Department of the City of New York.*** In 1871 the New York Department of Health became the first municipal agency in the United States to produce its own smallpox vaccine. Between 1876 and 1884 their vaccine farm was located in Clifton, NJ. Then it moved to New York City, where vaccine production continued in a vaccine farm located in the back of the Health Department on Mott Street [5] and in 1895 the vaccine laboratory was transferred to the Division of Pathology, Bacteriology and Disinfection [44]. Although the New York City Board of Health (NYCBH) vaccine stock was one of those selected in 1967 by the World Health Organization for its intensified global immunization campaign, the origin of the vaccine is obscure [45]. The original director of the vaccine farm, Dr. James R Taylor, reported that the stock was originally obtained from England, probably as far back as 1856, although other bovine viruses were also tried, although apparently not the Beaugency lymph. Other stocks used over the years may have included one from the Practical Institute for Animal Vaccine of Cuba and Puerto Rico [5]. This seed was used to initiate the production of ”animal vaccine” in 1871. On the other hand, a different vaccine strain obtained from England was apparently used for serial arm-to-arm vaccination. By 1876, these different smallpox vaccine seeds were used indistinctively, making the NYCBH strain probably a mix of several strains, as it occurred in many other vaccine producers of that time. Nevertheless, the so-called NYCBH strain is frequently referred to as the progenitor of all subsequent American smallpox vaccines, although the extant American vaccinia strains are most likely derived from multiple evolutionary events involving different stocks of 19th century vaccine viruses [46].

***National Vaccine and Antitoxin Institute.*** Despite its officially sounding name, it was a private organization founded around 1911 in Washington DC, and owned by Dwight T Scott. Its laboratory director was James Reverdy Steward, who in 1889 became a laboratory assistant to Theobald Smith in Harvard. Smith was a renowned German-born American microbiologist who had early and extensive contacts with Nobel laureate Paul Ehrlich regarding the production of diphtheria antitoxin, eventually establishing his own antitoxin and vaccine laboratory in Boston [47], [48].

## 5 What were the smallpox vaccine strains used during the turn of the 19–20 century in the United States?

Ever since Edward Jenner reported the preventative effect of cowpox against smallpox, the true nature of the vaccine has been the subject of great debate [3]. The debate continued in the United States at the beginning of the twentieth century, but this time it was not an abstract scientific discussion, but one focusing on the origin of the vaccine seeds used by the nascent smallpox vaccine industry [49]. In 1910 Milton Rosenau, the Director of the Hygienic Laboratory, U.S. Public Health and Marine-Hospital Service in Washington DC, pragmatically stated that the “vaccine virus is the specific principle in the material obtained from the skin eruption of calves having a disease known as vaccinia”, although he added that “the specific principle of vaccinia is unknown”. [50].

An early critic of vaccination was Dr. John W. Hodge, a medical doctor from Niagara Falls NY. In 1908 he explained that his opposition to vaccination was because he believed that smallpox could be prevented by having good health rather than “propagating the disease by vaccination”. He emphatically opposed vaccination because “it is not known what vaccine virus is, except that it is a mixed contagion of disease” [51]. In fact, Dr. Hodge had published the year before, in two homeopathic journals, his tribulations in trying to obtain information from the vaccine manufacturers about the nature of their smallpox vaccine [52], [53]. After unsuccessfully contacting several companies, he reported that only one response was received, from a representative from the Lancaster Vaccine Farms in Marietta. They informed that the late Dr. H. M. Alexander initially believed that their stock originated from a case of spontaneous cowpox that occurred in his farm, although he later believed that in fact it derived from a human case of smallpox. However, modern science refutes both interpretations because cowpox doesn’t exist in the Americas and cows are not susceptible to the variola virus.

A more credible piece of information about the source of the vaccine used by the vaccine farms came from the responses to a question sent in 1903 by the Journal of the American Medical Association [54]. Parke, Davis & Co. responded that their vaccines had been obtained from different sources, chiefly European, including the Imperial Vaccine Institute of Vienna and “the fine establishment” in Berne, Switzerland. The H. K. Mulford Co. stated that their vaccine virus is obtained from several sources, namely The Jenner Institute of Preventive Medicine in London, Chambon & Menard in Paris, Institute Vaccinale in Berne, Pasteur Institute in Lille, and from two unidentified sources in the United States. Although not revealing the source of his vaccine, Dr. H M. Alexander himself responded that he believed that vaccinia virus was simply smallpox passed through the cow. As for the NYCBH, a mixture of vaccine strains were probably used as described above.

A good indication of some of the preferred European sources of vaccine are the vaccine propagating establishments in Europe visited in 1903 by Dr. W. F. Elgin, the smallpox vaccine expert from H. K. Mulford Co, acting as a special Commissioner of the State Board of Health of Pennsylvania (Table 3) [55].

**Table 3:** Vaccine Propagating Establishments in Europe, visited by W.F. Elgin in 1903 [55].

Great Britain. Vaccine Department of the Local Government, London (F.R. Blaxall)
France. Institute Pasteur de Lille, Lille (M. Calmette)
France. Institute de Vaccine Animale, Paris (E. Chambon, Y. Menard)
Belgium. Vaccine Institute, Brussels (M. Degive)
Germany. Vaccine Institution, Central Meat Inspection Department, Berlin (Schutz)
Germany. Vaccine Institute, Cologne (Edward Mader)
Germany. Vaccine Institute, Dresden (Th. Chalybäas)
Switzerland. Vaccine Department, Serum and Vaccine Institute, Berne (A. Carini)
Switzerland. Vaccine Institute, Lausanne (E. Felix, J. Finck)
Italy. Vaccine Institute, Rome (Otavio Leoni)

As mentioned before, Dryvax from Wyeth, reputedly one of the oldest smallpox vaccines produced in the United States, is supposed to be derived from the New York City Board of Health (NYCBH) strain. However, information provided in 1886 by John Wyeth & Brother, states that “a Belgium establishment” was the origin of their vaccine, produced in Chester County [34]. The freeze-dried form of the Wyeth vaccine, Dryvax, was produced in the Marietta plant, after Wyeth’s acquisition of the Gilliland Laboratories in 1943.

Perhaps a response that honestly reflects a common practice during those early days was the one given by Robert Kennedy Cutter, son of the founder of the Cutter Laboratories, who in 1972 was asked for the origin of the seed used to manufacture the Cutter smallpox vaccine. His response was that they could have obtained it from any laboratory, even purchasing a vial of a vaccine from somebody else and using is as a starter [56]. In fact, despite the competition in the field of vaccines and other biological products, the laboratories frequently exchanged or purchased vaccine “seeds” from other laboratories.

## 6 Concluding remarks

It is remarkable that smallpox vaccination was developed and used for more than one century based on enlightened empiricism with no knowledge of the nature of viruses. Cowpox and horsepox were animal diseases and the matter from lesions was called “lymph”. For much of the 19th century the true nature of the preservative against smallpox was discussed among the medical community, although it was generally assumed to be derived from the cowpox or horsepox, animal diseases that only existed in Europe [57], [58]. Viruses were only identified as a novel group of microorganisms at the very end of the 19th century. The first animal virus described was the one that causes foot-and-mouth disease, by Loeffler and Frosch in 1898 in Berlin, and in 1905 the Italian pathologist Adelchi Negri proved that the vaccinia virus passes through bacteriological filers [18]. It was not until 1939 when Allan Watt Downie, a Professor of bacteriology in the University of Liverpool, used serological techniques to demonstrate that the virus used at that time for vaccination against smallpox, now referred to as vaccinia virus, was different from what was known as cowpox virus [59]. That discovery reopened the scientific discussion about the true origin of the vaccinia virus [60]. Since a natural animal host for vaccinia virus has not been identified, vaccinia is often referred to as a laboratory virus that could have originated from a still unidentified animal Orthopoxvirus ancestor. Derrick Baxby, then a lecturer in Medical Microbiology at the University of Liverpool, studied the historical record reaching the conclusion that Jenner’s original interpretation was correct, that a hypothetical horsepox virus could be the long sought ancestor of vaccinia [61]. In fact, we recently reviewed the extensive use of equination (inoculation of the horsepox) as an alternative to vaccination (inoculation of the cowpox) during the 19th century [3]. Several investigators have reported that contemporary vaccinia strains are variants of related viruses. The recognition of the polyclonal and shifting nature of vaccine variants led the United States to adopt a clonal vaccine, ACAM2000, which was derived from Dryvax and is grown in cell culture [62].

Orthopoxviruses have low mutation rates in culture, even after serial passages when deletions, transversions and duplications are observed to a greater extent than point mutations. It is the case of the strain Chorioallantois vaccinia virus Ankara (CVA) passed over 500 times in avian cells resulting in the Modified Vaccinia Ankara (strain MVA), which has numerous deletions more than point mutations [63]. Similar events may have happened as a consequence of the serial passage of different smallpox vaccine strains in cows, leading to polyclonal vaccine stocks. Therefore, it is reasonable to assume that the polyclonal nature of cultivated smallpox vaccine “strains” is the legacy of mixing of different vaccine strains into artificial preparations that likely occurred during the industrialization of smallpox vaccines manufacturing in cows.

While mutation rates are low, co-infection of vaccine strains results in a reasonably high rate of homologous recombination. Analysis of individual clones from smallpox vaccines, such as the Brazilian IOC strain and Dryvax in particular, indicate that the clones appear to represent viruses derived from complex recombinational events between different strains of vaccinia virus that appear to have included a common ancestor with horsepox virus [36], [46], [64].

The industrialization of manufacturing animal vaccine by “Vaccine Farms” in the U.S. and “Vaccine Parks” in Europe appears to have led to adaptation of Jenner’s original vaccine virus for growth in cows. Modern sequence analysis of vaccinia strains from around the world show similar, but different deletions in the regions adjacent to the Left and Right Inverted Terminal Repeats (ITRs). These regions are known to encode genes involved in host range and that encode proteins that interact with components of the host immune system. Taken together, the passage of vaccinia in cows over more than 100 years has led to an array of modern vaccinia strains and variants that have convergently evolved to delete segments adjacent to the Left and Right ITRs.

We recently reported that a smallpox vaccine commercially produced in the United States in 1902 by H. K. Mulford is closely related to the only sequence yet identified for modern (1976) horsepox, strongly supporting the hypothesis that at least some of the early vaccines used to protect against smallpox were based on viruses that would now be called horsepox virus [65].

The historical information reviewed in this article provides a framework to help interpreting the results from our current efforts to sequence and characterize a number of early smallpox vaccines manufactured in the United States and in other countries in the Americas, Europe and Asia, with the goal of better understanding the origin and evolution of the vaccines used to finally eradicate the smallpox in 1980 [4].

## Authorship

All authors attest they meet the ICMJE criteria for authorship. JE conceptualized and wrote the article. SL, AN, and CD provided information and critically analyzed and contributed to the manuscript.

## Funding

The preparation of this article was partially supported by a grant from the 10.13039/100000865Bill & Melinda Gates Foundation, United States (OPP1216026). CRD received support from the 10.13039/501100003593Brazilian Conselho Nacional de Desenvolvimiento Científico e Tecnológico (CNPq), 10.13039/501100002322Coordenação de Aperfeiçoamento de Pessoal de Nivel Superior (CAPES) and 10.13039/501100004586Fundação Carlos Chagas Filho de Amparo à Pesquisa do Estado do Rio de Janeiro (FAPERJ), Brazil. AN received support by the 10.13039/501100005890German Ministry of Health, Germany.

## Declaration of Competing Interest

The authors declare that they have no known competing financial interests or personal relationships that could have appeared to influence the work reported in this paper.
